# Effects of astragaloside IV on the pharmacokinetics of omeprazole in rats

**DOI:** 10.1080/13880209.2019.1636828

**Published:** 2019-07-10

**Authors:** Wei Liu, Guozhi Liu, Jing Liu

**Affiliations:** a Department of Pediatric Medicine, Yidu Central Hospital of Weifang, Weifang, China;; b Department of Neonatology, Yidu Central Hospital of Weifang, Weifang, China

**Keywords:** CYP3A4, herb–drug interaction, P-gp

## Abstract

**Context:** Omeprazole and astragaloside IV (AS-IV) are widely used for the treatment of gastric ulcers in China clinics.

**Objective:** This study investigates the effects of AS-IV on the pharmacokinetics of omeprazole in rats.

**Materials and methods:** The pharmacokinetics of orally administered omeprazole (2 mg/kg), with or without AS-IV (100 mg/kg/day for 7 days) pretreatment, were investigated in male Sprague-Dawley rats (two groups of six animals each) using LC–MS/MS. A Caco-2 cell transwell model and rat liver microsome incubation systems were also used to support the *in vivo* pharmacokinetic data and investigate its potential mechanism.

**Results:** The results indicated that co-administration of AS-IV could decrease the systemic exposure of omeprazole significantly (*p* < 0.05), including AUC_0–_*_t_* (717.20 ± 177.63 vs. 1166.25 ± 186.65 ng h/mL) and *C*_max_ (272.35 ± 25.81 vs. 366.34 ± 32.57 ng/mL). The *t*_1/2_ of omeprazole also decreased significantly (1.78 ± 0.15 vs. 2.23 ± 0.27 h, *p* < 0.05). The efflux ratio of omeprazole across the Caco-2 cell transwell model increased significantly from 1.73 to 2.67 (*p* < 0.05), and the metabolic stability of omeprazole was decreased from 42.6 ± 7.8 to 26.2 ± 5.1 min with the pretreatment of AS-IV (*p* < 0.05).

**Discussion and conclusions:** AS-IV could decrease the systemic exposure of omeprazole in rats when AS-IV and omeprazole were co-administered, and it might exert these effects through decreasing the absorption of omeprazole by inducing *P-gp*, or through accelerating the metabolism of omeprazole in rat liver by inducing the activity of CYP3A4.

## Introduction

Omeprazole is an atypical proton pump inhibitor. It is prescribed for the treatment of various acid-related diseases, such as peptic ulcer, gastroesophageal reflux diseases (Herrera-Mozo et al. 2017; Selmi et al. 2017; Talaat 2017; Yang et al. 2017). Omeprazole can be rapidly absorbed from the gut lumen, and however, the oral bioavailability of omeprazole is low. Fang et al. (2009) have reported that oral omeprazole undergoes marked extraction in the small intestine, and i.d. administration of ketoconazole and verapamil could increase the bioavailability of omeprazole through inhibiting the function of CYP3A4 and P-gp. Consequently, co-administration of foods, drugs or dietary supplements with influence on CYP3A and/or P-gp may affect the pharmacokinetics of omeprazole, causing undesirable toxicity and/or diminishment of drug efficacy (Zhang et al. 2017; Zhao et al. 2017).

Radix astragali [the root of *Astragalus membranaceus* (Fish) Bungevar. Mongholicus (Bunge) Hsiao or *Astragalus membranaceus* (Fisch.) Bge. (Fabaceae)] has been used as one of the primary tonic herbs in traditional Chinese and Japanese Kampo medicine (Deng et al. 2016; Zhao et al. 2017). Astragaloside IV (AS-IV) is one of the major active compounds of Radix Astragali. AS-IV possesses a number of pharmacological effects, including immunoregulatory, anti-hypertensive, anti-oxidative, anti-inflammatory and anti-tumour effect (Zhang et al. 2003, 2006, 2011; Luo et al. 2004; Li et al. 2016). Several research articles have indicated that AS-IV could modulate the activity of CYP3A4 and *P-gp*, which might lead to drug–drug interactions when they are co-administered with other herbs or drugs (Wang et al. 2014; Zhang et al. 2016). As we know, omeprazole and AS-IV are widely used for the treatment of peptic ulcer in China clinics, and however, no reports were found on possible pharmacokinetic interactions between AS-IV and omeprazole, especially the effects of AS-IV on the pharmacokinetics of omeprazole.

This study investigates the effects of AS-IV on the oral pharmacokinetics of omeprazole in rats and to explore the possible mechanisms of herb–drug interaction using Caco-2 cell transwell model and rat liver microsomes incubation systems.

## Materials and methods

### Chemicals and reagents

Omeprazole (purity >98%), AS-IV (purity >98%) and esculin (purity >98%) were purchased from the National Institute for the Control of Pharmaceutical and Biological Products (Beijing, China). Rat liver microsomes were purchased from BD Gentest (Woburn, MA, USA). Dulbecco’s modified Eagle’s medium (DMEM) and non-essential amino acid (NEAA) solution were purchased from Thermo Scientific Corp. (Logan, UT, USA). Acetonitrile and methanol were purchased from Fisher Scientific (Fair Lawn, NJ, USA). Formic acid was purchased from Anaqua Chemicals Supply Inc. Limited (Houston, TX, USA). Ultrapure water was prepared with a Milli-Q water purification system (Billerica, MA, USA). All other chemicals were of analytical grade or better.

### Animal experiments

Male Sprague-Dawley (SD) rats weighing 220–250 g were provided by the Shanghai University of TCM (Shanghai, China). The animal care, use and experimental protocols were also approved by the animal care committee of the Shanghai University of TCM (Shanghai, China). Rats were bred in a breeding room at 25 °C with 60 ± 5% humidity and a 12 h dark/light cycle. Tap water and normal chow were given *ad libitum*. All of the experimental animals were housed under the above conditions for a three-day acclimation period and fasted overnight before the experiments.

### LC–MS/MS determination of omeprazole

The determination was performed on an Agilent 1290 series liquid chromatography system and an Agilent 6470 triple-quadruple mass spectrometer (Palo Alto, CA, USA). The chromatographic analysis of omeprazole was performed on a Waters Xbridge C18 column (3.0 × 100 mm, i.d.; 3.5 μm) at room temperature. The mobile phase was water (containing 0.1% formic acid) and acetonitrile (25:75, v:v) at a flow rate of 0.3 mL/min. The mass scan mode was positive MRM mode. The precursor ion and product ion are *m*/*z* 345.8 → 197.7 for omeprazole and *m*/*z* 339.0 → 176.9 for esculin, respectively. The collision energy for omeprazole and IS was 20 and 35 eV, respectively. The MS/MS conditions were optimized as follows: fragmentor, 110 V; capillary voltage, 3.5 kV; nozzle voltage, 500 V; nebulizer gas pressure (N_2_), 40 psig; drying gas flow (N_2_), 10 L/min; gas temperature, 350 °C; sheath gas temperature, 400 °C; sheath gas flow, 11 L/min.

### In vivo pharmacokinetic study

To evaluate the effects of AS-IV on the pharmacokinetics of omeprazole, the rats were divided into two groups (group A and group B) of six animals each. Group A: treated with omeprazole (orally administered to rats at a dose of 2 mg/kg) alone (Sykes et al. 2015); Group B: co-administration of omeprazole (2 mg/kg) with the pretreatment of AS-IV (100 mg/kg/day for 7 days) (Liu et al. 2018). Blood samples (250 μL) were collected into heparinized tubes *via* the *oculi chorioideae* vein at 0.083, 0.167, 0.33, 0.5, 1, 2, 4, 6, 8, 12 and 24 h after the omeprazole treatment, respectively. The blood samples were centrifuged at 3000 rpm for 5 min, and the plasma samples obtained were stored at −40 °C until the analysis.

### Data analysis

The pharmacokinetic parameters were calculated using the DAS 3.0 pharmacokinetic software (Chinese Pharmacological Association, Anhui, China). The differences between the mean values were analyzed for significance using a one-way analysis of variance (ANOVA). Values of *p <* 0.05 were considered to be statistically significant.

### Cell culture

The Caco-2 cell line was obtained from the American Type Culture Collection (Manassas, VA, USA). The Caco-2 cells were cultured in DMEM high glucose medium containing 15% FBS, 1% NEAA and 100 U/mL penicillin and streptomycin. The cells were cultured at 37 °C with 5% CO_2_. For transport studies, the cells at passage 40 were seeded on transwell polycarbonate insert filters (1.12 cm^2^ surface, 0.4 μm pore size, 12 mm diameter) (Cambridge, MA, USA) in 12-well plates at a density of 1 × 10^5^ cells/cm^2^. Cells were allowed to grow for 21 days. For the first seven days, the medium was replaced every two days, and then daily. The transepithelial electrical resistance (TEER) of the monolayer cells was measured using Millicell ERS-2 (Billerica, MA, USA), and TEER exceeding 400 Ω cm^2^ was used for the flux experiment. The integrity of the Caco-2 monolayers was confirmed by the paracellular flux of Lucifer yellow, which was less than 1% per hour. The alkaline phosphatase activity was validated using an Alkaline Phosphatase Assay Kit. The qualified monolayers were used for transport studies.

### Effects of As-IV on the absorption of omeprazole in Caco-2 cell transwell model

Before the transport experiments, the cell monolayers were rinsed twice using warm (37 °C) Hanks’ balanced salt solution (HBSS), then the cells were incubated at 37 °C for 20 min. After preincubation, the cell monolayers were incubated with omeprazole in fresh incubation medium added on either the apical or basolateral side for the indicated times at 37 °C. The volume of incubation medium on the apical and basolateral sides was 0.5 mL and 1.5 mL, respectively, and a 100 μL aliquot of the incubation solution was withdrawn at the indicated time points from the receiver compartment and replaced with the same volume of fresh pre-warmed HBSS buffer. The effects of AS-IV on the omeprazole flux by Caco-2 cells were investigated by adding AS-IV (50 μM) to both sides of the cell monolayers and preincubating the sample at 37 °C for 30 min. The permeability of omeprazole (10 μM) in all of the above conditions for both directions, that is, from the apical (AP) side to the basolateral (BL) side and from the BL side to the AP side, was measured after incubation for 30, 60, 90 and 120 min at 37 °C.

The apparent permeability coefficient (*P*_app_) was calculated using the equation of Artursson and Karlsson:

$$P_{\text{app}}=\;\left(\Delta Q/\Delta t\right)\;\times \;\left[1/\left(A\;\times \;C_{0}\right)\right]$$

where *P*_app_ is the apparent permeability coefficient (cm/s), Δ*Q*/Δ*t* (μmol/s) is the rate at which the compound appears in the receiver chamber, *C*_0_ (μmol/L) is the initial concentration of the compound in the donor chamber and *A* (cm^2^) represents the surface area of the cell monolayer. Data were collected from three separate experiments, and each was performed in triplicate.

### Effects of As-IV on the metabolic stability of omeprazole in rat liver microsomes

The effects of AS-IV on the metabolic stability of omeprazole were investigated using rat liver microsome incubation experiments. The assay conditions and reaction mixtures were similar to those reported previously (Li et al. 2016; Wang et al. 2017). In brief, except for NADPH-generating system, 10 μL rat liver microsomes (20 mg/mL), 4 μL omeprazole solution (100 μM) and 366 μL PBS buffer were added to the centrifuge tubes on ice. The reaction mixture was incubated at 37 °C for 5 min and then NADPH-generating system (15 μL) was added. The effects of AS-IV on the metabolic stability of omeprazole were investigated by adding 5 mM of AS-IV (final concentration of 50 μM) to rat liver microsomes and preincubating them for 30 min at 37 °C, followed by the addition of omeprazole (final concentration of 1 μM). Aliquots of 30 μL were collected from reaction volumes at 0, 1, 3, 5, 15, 30 and 60 min and 60 μL ice-cold acetonitrile containing IS was added to terminate the reaction, and then the concentration of omeprazole was determined using LC–MS/MS.

The *in vitro half-life* (*t*_1/2_) was obtained using the equation: *t*_1/2_ = 0.693/k.

## Results and discussion

### Effects of As-IV on the pharmacokinetics of omeprazole

**Figure 1.**
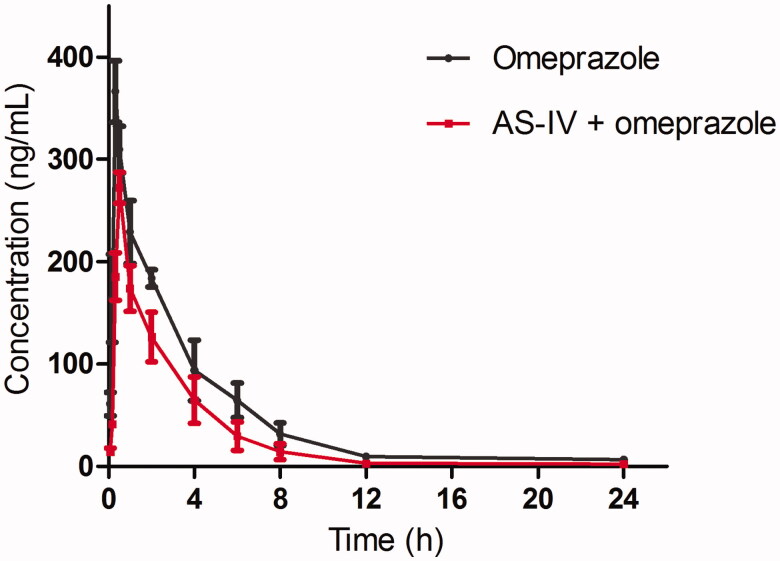
The pharmacokinetic profiles of omeprazole in rats (six rats in each group) after the oral administration of omeprazole (2 mg/kg) with or without AS-IV pretreatment (100 mg/kg/day for 7 days). Each point represents the average ± S.D. of six determinations.

The pharmacokinetic parameters are shown in Table 1. As shown in Figure 1, when the rats were pretreated with AS-IV, the *C*_max_ of omeprazole was decreased from 366.34 ± 32.57 to 272.35 ± 25.81 ng/mL, and the difference was significant (*p* < 0.05). The AUC_(0–inf)_ omeprazole was also significantly lower than that of the control (*p* < 0.05). However, the *T*_max_ of omeprazole increased from 0.29 ± 0.03 to 0.50 ± 0.06 h, and the difference was significant (*p* < 0.05). The *t*_1/2_ of omeprazole in rats pretreated with AS-IV was decreased compared with the control (1.78 ± 0.15 vs. 2.23 ± 0.27 h), and the difference was also significant (*p* < 0.05).

**Table 1.** Pharmacokinetic parameter of omeprazole in rats after oral administration of omeprazole (2 mg/kg; *n* = 6, Mean ± SD) with or without treatment of AS-IV (100 mg/kg/day for 7 days).

Parameter	Omeprazole	AS-IV +
*T*_max_ (h)	0.29 ± 0.03	0.50 ± 0.06*
*C*_max_ (ng/mL)	366.34 ± 32.57	272.35 ± 25.81*
*t*_1/2_ (h)	2.23 ± 0.27	1.78 ± 0.15*
AUC_(0–inf)_ (ng h/mL)	1166.25 ± 186.65	717.20 ± 177.63*

* *p* < 0.05 indicates significant differences from the control.

The results indicated that AS-IV could decrease the systemic exposure of omeprazole in rats when AS-IV and omeprazole were co-administered. Therefore, we think that the herb–drug interaction between AS-IV and omeprazole should be cautioned when they are co-administered. However, due to the pharmacokinetic differences of human and rats, further *in vivo* system studies are needed to identify the interactions in humans.

Some other research also found that when AS-IV was co-administered with other drugs, their bioavailability would be decreased (Song et al. 2014a, 2014b; Liu et al. 2018). As AS-IV has been reported to induce the activity of P-gp and CYP3A4, we speculated that AS-IV might decrease the absorption of omeprazole by inducing P-gp-mediated drug efflux or CYP3A4-mediated metabolism.

### Effects of As-IV on the bidirectional transport of omeprazole across Caco-2 cells

To investigate the effects of AS-IV on the transport of omeprazole, the Caco-2 cell transwell model was utilized. The *P*_appAB_ and *P*_appBA_ were 2.06 ± 0.22 × 10^−6^ and 3.57 ± 0.41 × 10^−6 ^cm/s, respectively. The *P*_appBA_ was much higher than the *P*_appAB_. Then, the transport studies were performed in the presence of AS-IV. In the presence of AS-IV, the *P*_app_ values from the AP side to the BL side decreased (1.52 ± 0.18 × 10^−6 ^cm/s), whereas those from the BL side to the AP side decreased (4.06 ± 0.55 × 10^−6 ^cm/s). The efflux ratio increased from 1.73 to 2.67, and the absorption of omeprazole was decreased significantly. Fang et al. (2009) have reported when co-administered with verapamil, an typical P-gp inhibitor, the absorption of omeprazole will be increased. As AS-IV could induce the activity of P-gp, and therefore, we think that AS-IV might decrease the absorption of omeprazole through inducing the activity of P-gp.

### Effects of As-IV on the metabolic stability of omeprazole in rat liver microsomes

The effects of AS-IV on the metabolic stability of omeprazole were investigated using rat liver microsomes. The metabolic half-life of omeprazole was 42.6 ± 7.8 min, while the metabolic half-life was prolonged (26.2 ± 5.1 min) in the presence of AS-IV, and the difference was significant (*p* < 0.05). These results indicated that AS-IV could accelerate the metabolism of omeprazole in rat liver microsomes *via* inducing the activity of CYP3A4. These results suggested that AS-IV might increase the metabolic clearance through inducing the activity of CYP3A4.

## Conclusions

In conclusion, AS-IV could decrease the systemic exposure of omeprazole in rats when AS-IV and omeprazole were co-administered. We infer that AS-IV might exert these effects mainly through decreasing the absorption of omeprazole by inducing the activity of *P-gp*, and or through accelerating the metabolism of omeprazole in rat liver by inducing the activity of CYP3A4. Therefore, we think that the herb–drug interaction between AS-IV and omeprazole might occur when they are co-administered.
